# MIEAP and ATG5 are tumor suppressors in a mouse model of BRAF^V600E^-positive thyroid cancer

**DOI:** 10.3389/fendo.2022.932754

**Published:** 2022-09-15

**Authors:** Koichiro Hamada, Tomomi Kurashige, Mika Shimamura, Hirofumi Arakawa, Yasuyuki Nakamura, Yuji Nagayama

**Affiliations:** ^1^ Department of Molecular Medicine, Atomic Bomb Disease Institute and Nagasaki University Graduate School of Biomedical Sciences, Nagasaki, Japan; ^2^ Department of General Medicine, Nagasaki University Graduate School of Biomedical Sciences, Nagasaki, Japan; ^3^ Division of Cancer Biology, National Cancer Center Research Institute, Tokyo, Japan

**Keywords:** thyroid cancer, ATG5, MIEAP, oncocytoma, mitochondria

## Abstract

Mitochondria-eating protein (MIEAP) is a molecule important for non-canonical mitophagy and thought to be a tumor suppressor. Our previous study found that MIEAP expression is defective in thyroid oncocytomas, irrespective of being benign or malignant, and also in non-oncocytic thyroid cancers. Thyroid oncocytomas are composed of large polygonal cells with eosinophilic cytoplasm that is rich in abnormal mitochondria. Thus, our data indicate that, together with increased mitochondrial biogenesis that compensates for the dysfunction of the mitochondria, MIEAP plays a critical role in the accumulation of mitochondria in thyroid oncocytic tumors, whereas a defective MIEAP expression alone is not sufficient for mitochondrial accumulation in non-oncocytic cancers with normal mitochondria. To clarify whether MIEAP is a tumor suppressor in the thyroids and whether MIEAP knockout (KO) alone is sufficient for the oncocytic phenotype and also to extend our effort toward canonical mitophagy (a selective autophagy), we here conducted mouse studies using genetically engineered mice. *Braf^CA/wt^* mice developed thyroid cancers 1 year after intrathyroidal injection of adenovirus expressing Cre, while cancer development was observed at 6 months in adenovirus-Cre-injected *Braf^CA/wt^;Mieap^KO/KO^* and *Braf^CA/wt^;Atg5^flox/flox^* mice [where autophagy-related 5 (ATG5) is a component of autophagic machinery], although KO of either molecule alone was not sufficient for cancer development. These data demonstrate that MIEAP or ATG5 KO accelerated thyroid cancer development. However, cancers in adenovirus-Cre-injected *Braf^CA/wt^*;*Mieap^KO/KO^* and *Braf^CA/wt^*;*Atg5^flox/flox^* mice were not oncocytic. In conclusion, we here show that MIEAP and ATG5 are both tumor suppressors in thyroid carcinogenesis, but as we have anticipated from our previous data, KO of either molecule does not confer the oncocytic phenotype to BRAF^V600E^-positive thyroid cancers. The combination of disruptive mitochondrial function and impaired mitochondrial quality control may be necessary to establish a mouse model of thyroid oncocytoma.

## Introduction

Thyroid oncocytic tumors (also called eosinophilic or oxyphilic tumors) are characterized by large polygonal cells with eosinophilic cytoplasm (1). These tumors contain abnormally enlarged mitochondria in excess and frequently have mutations in mitochondrial DNA (mtDNA) encoding complex I of the electron transport chain (2). Therefore, the mitochondria accumulated are dysfunctional.

Although it has previously been reported that mitochondrial accumulation is due to compensated increase in mitochondrial biogenesis (3–6), abnormal mitochondria are usually eliminated by a mitochondrial quality control system. Mitophagy, a type of selective autophagy, is included in this system (hereafter called “canonical mitophagy”). A thyroid oncocytic carcinoma cell line XTC.UC1 (7) has been found to have a loss–of–function mutation (V380L) in PARK2, causing defective canonical mitophagy (8). However, the same mutation exists only in one of seven oncocytic cell carcinomas, and no mutations have been found in genes coding the components of canonical mitophagy machinery in thyroid oncocytic carcinomas (9–11).

Recently, another mitochondrial quality control system has been found, in which mitochondria–eating protein (MIEAP) plays an indispensable role (called “non–canonical mitophagy”) (12). The expression of MIEAP has been reported to be suppressed by the methylation of its promoter in a fraction of colon, breast, and gastric cancers (13–16). Gastric cancer cell lines with defective MEAP expression are more invasive in a hypoxic condition than those with intact MIEAP expression (17), and gastric and breast cancer cell lines overexpressing MIEAP die through apoptosis (14, 15). Breast cancer patients with a higher MIEAP expression have a better prognosis than those with a lower expression (16). Furthermore, in a mouse model, *Apc^Min/+^,Mieap^KO/KO^* mice show more aggressive intestinal lesions than *Apc^Min/+^* mice (18). Thus, MIEAP is now thought to be a tumor suppressor.

We have recently found that MIEAP was expressed in human thyroid non–oncocytic (conventional) follicular adenomas (FAs), but not in oncocytic FAs and conventional and oncocytic carcinomas (19), and therefore postulated that loss of MIEAP expression and a compensatory increase in mitochondria biogenesis induce the accumulation of mitochondria in thyroid oncocytic adenomas and cancers, however, in thyroid conventional cancers, the mitochondria are not accumulated despite defective MIEAP expression because of no compensatory increase in mitochondrial biogenesis. Functionally, in XTC.UC1 cells in which the expression of MIEAP is also suppressed, exogenously induced MIEAP reduced the amounts of abnormal mitochondria, as indicated by decreased reactive oxygen species levels, mtDNA/nuclear DNA ratios, and cytoplasmic acidification.

To further explore the roles of MIEAP in the oncocytic phenotype of thyroid tumors and in the carcinogenesis of thyroid cancers in general (that is, both oncocytic and conventional cancers) as a tumor suppressor, we utilized a mouse model of BRAF^V600E^–positive thyroid cancer that we have previously established. In this model, *Braf^CA^* mice developed thyroid cancers 1 year after intrathyroidal injection of adenovirus expressing Cre DNA recombinase (20). MIEAP–defective *Braf^CA^* mice were generated (*Braf^CA/wt^,Mieap^KO/KO^*), and their thyroid tumorigenicity and oncocytic phenotype were compared with those in *Braf^CA/wt^* and *Mieap^KO/KO^* mice. Moreover, to extend our effort toward the study on the role of canonical autophagy in thyroid carcinogenesis, the conditional autophagy–related 5 (ATG5)–defective (*Atg5^flox/flox^*) mice were also employed and used to generate *Braf^CA/wt^*,*Atg5^flox/flox^* mice.

## Materials and methods

### Generation of Braf^CA/wt^,Mieap^KO/KO^ and Braf^CA/wt^,Atg5^flox/flox^ mice

*Braf^CA^* (B6.129P2(Cg)–*Braf^tm1Mmcm^*/J, stock# 017837) mice (21), having Cre–activating *Braf^V600E^*, were obtained from Jackson Laboratory (Bar Harbor, ME, USA). *Mieap^KO^* mice have previously been generated (18). *Atg5^flox^* mice, in which exon 3 of the *Atg5* gene is flanked by two *loxP* sequences, have originally been generated by N. Mizushima (22) and were provided through the RIKEN Bioresource Center (Tsukuba, Japan, http://en.brc.riken.jp/).

*Braf^CA/wt^* mice were crossed with *Mieap^KO/KO^* or *Atg5^flox/flox^*, generating *Braf^CA/wt^,Mieap^KO/wt^* or *Braf^CA/wt^,Atg5^flox/wt^* mice, which were subsequently backcrossed to *Mieap^KO/KO^* or *Atg5^flox/flox^* mice to produce *Braf^CA/wt^,Mieap^KO/KO^* or *Braf^CA/wt^,Atg5^flox/flox^* mice, respectively. Upon intrathyroidal Cre DNA recombinase expression (that is, injection of Ad–TgP–Cre (23) in our case, see below), the thyroids of these mice express BRAF^V600E^ and lose ATG5 expression.

### Experimental designs

Surgery and injection of adenovirus into the thyroid left lobes of 4– to 6–week–old mice were performed as previously described (20, 23). The adenovirus used in this study was Ad–TgP–Cre, in which Cre DNA recombinase is expressed under the control of a thyroid–specific thyroglobulin (Tg) promoter (23). A total of 3–4 × 10^9^ adenovirus particles/mouse were injected. Ad–TgP–Cre–injected *Braf^CA/wt^* and *Atg5^flox/flox^* mice were designated as *Braf^thyr–V600E^* and *Atg5^thyr–KO/KO^* mice, respectively. The male–to–female mice ratio was approximately 1:1 in all the experimental groups. After 6 and 12 months, mice were anesthetized with isoflurane, blood was collected *via* cardiac tap, and mice were euthanized by cervical dislocation. For histological examinations, the thyroids were removed from all mice.

Animal care and all experimental procedures were performed in accordance with the Guideline for Animal Experimentation of Nagasaki University with the approval of the Institutional Animal Care and Use Committee (permission number: 1803191437). All surgeries were performed under isoflurane anesthesia, and every effort was made to minimize suffering.

### Hematoxylin and eosin staining and immunohistochemistry

Tissues were fixed in 10% neutral–buffered formalin and then embedded in paraffin. Sections (4 μm thick) were prepared and stained with hematoxylin and eosin (H&E) or immunostained with primary antibodies: rabbit monoclonal anti–Tg (ab156008, Abcam, Cambridge, UK, dilution of 1:250), rabbit monoclonal anti–Ki–67 (ab66155, Abcam, dilution of 1:100), rabbit polyclonal anti–tumor suppressor P53–binding protein 1 (53BP1) (A300–272A, Bethyl Laboratories, Montgomery, TX, USA, dilution of 1:200), rabbit monoclonal anti–MIEAP (ab180154, Abcam, dilution of 1:400), and rabbit polyclonal anti–TOMM20 (translocase of outer mitochondrial membrane 20) (11802–1–AP, Proteintech, Tokyo, Japan, dilution of 1:250). Immunohistochemistry was performed with an ImmPRESS^®^ HRP horse anti–rabbit IgG PLUS Polymer Kit, Peroxidase (MP–7801, Vector Laboratories, Burlingame, CA, USA) for Tg and Ki–67 and the 488–conjugated goat polyclonal anti–rabbit IgG (A11008, Life Technologies, Tokyo, Japan, dilution of 1:200) for 53BP1, MIEAP, and TOMM20. Slides were analyzed using an All–in–One BZ–9000 Fluorescence Microscope (Keyence, Osaka, Japan), and the fluorescent intensity was quantified using a BZ–II Analyzer (Keyence). At least 100 cells were evaluated in each sample to measure the fluorescent intensities of TOMM20 and the number of Ki–67 and 53BP1 foci.

### Evaluation of apoptosis

TUNEL staining was performed with an Apop–tag™ Fluorescein Direct *in situ* Apoptosis Detection Kit (Merck Millipore, Darmstadt, Germany). The slides were embedded with a VECTASHIELD Mounting Medium containing 4′,6–diamidino–2–phenylindole (Vector Laboratories, Burlingame, CA, USA) and analyzed using an All–in–One BZ–9000 Fluorescence Microscope. At least 100 cells were evaluated in each sample to determine the percentages of TUNEL–positive cells.

### Evaluation of the prognostic significance of MIEAP and ATG5

The prognostic values of MIEAP and ATG5 expression levels were evaluated with The Human Protein Atlas (https://www.proteinatlas.org/), which is connected to The Cancer Genome Atlas (TCGA) dataset (https://www.cancer.gov/about–nci/organization/ccg/research/structural–genomics/tcga).

### Statistical analyses

All data were expressed as means ± or SE and analyzed for significant differences using the Student’s *t*–test. A *P*–value of less than 0.05 was considered statistically significant.

## Results

**Figure 1:**
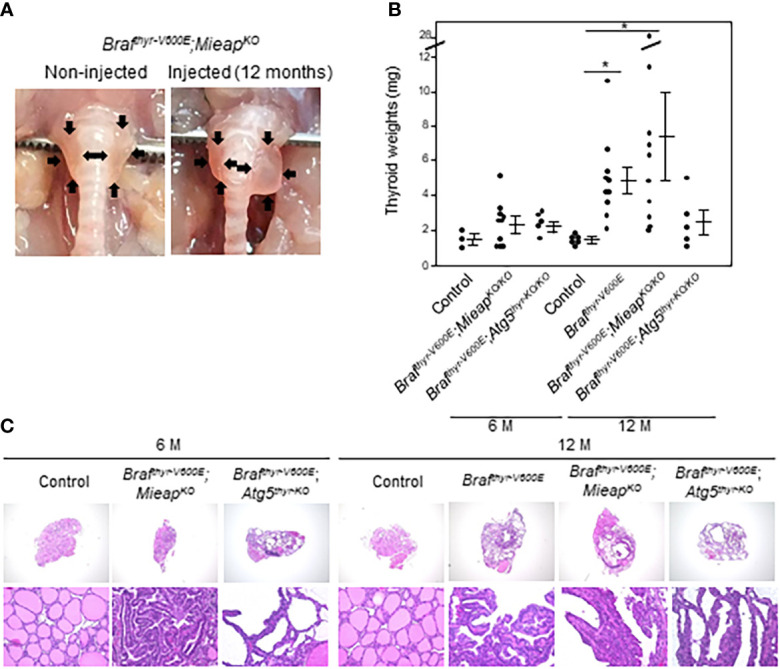
Gross appearance **(A)**, thyroid left lobe weights **(B)**, and histology **(C)** in control, *Braf^thyr–V600E^*, *Braf^thyr–V600E^*,*Mieap^KO^*, and *Braf^thyr–V600E^*,*Atg5^thyr–KO/KO^* mice. Mice were sacrificed at 6 and 12 months. The thyroid glands were removed, and thyroid weights and histology were determined as described in *Materials and methods*. Data are shown as black circles for individual mice and as means ± SE. *P < 0.05. M, months. The original magnifications were ×40 and ×400.

To investigate the role of MIEAP and ATG5 in thyroid carcinogenesis, we prepared *Braf^thyr–V600E^*, *Mieap^KO/KO^*, *Atg5^thyr–KO/KO^*, *Braf^thyr–V600E^*,*Mieap^KO/KO^*, and *Braf^thyr–V600E^*,*Atg5^thyr–KO/KO^* mice. Mice were subjected to intrathyroidal injection of Ad–TgP–Cre at 4 to 6 weeks and sacrificed at 6 and 12 months. Macroscopically, the thyroids of *Mieap^KO/KO^* and *Atg5^thyr–KO/KO^* mice were normal at 6 and 12 months, and those of most of *Braf^thyr–V600E^* mice were enlarged at 12, but not 6, months as we have previously reported (20). In *Braf^thyr–V600E^*,*Mieap^KO/KO^* and *Braf^thyr–V600E^*,*Atg5^thyr–KO/KO^* mice, thyroid enlargements were detected at not only 12 but also 6 months (**Table 1**). The representative pictures of the thyroids in non–injected and injected *Braf^thyr–V600E^*,*Mieap^KO/KO^* mice at 12 months are shown in **Figure 1A**. The thyroid weights of each mouse group are shown in **Figure 1B**. There was no difference in the thyroid left lobe weights among the control, *Braf^thyr–V600E^*,*Mieap^KO/KO^*, and *Braf^thyr–V600E^*,*Atg5^thyr–KO/KO^* mice at 6 months, but the injected left lobes were significantly heavier in *Braf^thyr–V600E^* and *Braf^thyr–V600E^*,*Mieap^–KO/KO^* mice than the control at 12 months (**Figure 1B**). The injected left lobes of *Braf^thyr–V600E^*,*Atg5^thyr–KO/KO^* mice tended to be only slightly (but not significantly) heavier than the control lobes.

**Table 1:** Summary of incidences of thyroid tumors in Braf^thyr–V600E^, Mieap^KO/KO^, Atg5^thyr–KO/KO^, Braf^thyr–V600E^,Mieap^KO/KO^, and Braf^thyr–V600E^,Atg5^thyr–KO/KO^ mice.

Mice	Incidence of tumors	
	6 months	12 months
*Braf^thyr–V600E^*	0/10	9/10
*Mieap^KO/KO^*	0/10	0/10
*Atg5^thyr–KO/KO^*	0/8	0/15
*Braf^thyr–V600E^,Mieap^KO/KO^*	7/10	9/10
*Braf^thyr–V600E^,Atg5^thyr–KO/KO^*	9/10	10/12

In H&E staining, all the enlarged thyroid left lobes of *Braf^thyr–V600E^* mice at 12 months and of *Braf^thyr–V600E^*,*Mieap^KO/KO^* and *Braf^thyr–V600E^*,*Atg5^thyr–V600E^* mice at 6 and 12 months comparably showed papillary growth of atypical follicular cells exhibiting hyperchromatic irregular–shaped nuclei and intranuclear cytoplasmic inclusions and follicular and nestic growth of atypical follicular cells with severe nuclear atypia as we have previously detected in *Braf^thyr–V600E^* mice (20) (**Figure 1C** and data not shown). Again, the thyroids of *Mieap^KO/KO^* and *Atg5^thyr–KO/KO^* mice were normal at 6 and 12 months. Thus, *Braf^thyr–V600E^*,*Mieap^KO/KO^* and *Braf^thyr–V600E^*,*Atg5^thyr–KO/KO^* mice developed thyroid cancers earlier than *Braf^thyr–V600E^* mice.

**Figure 2:**
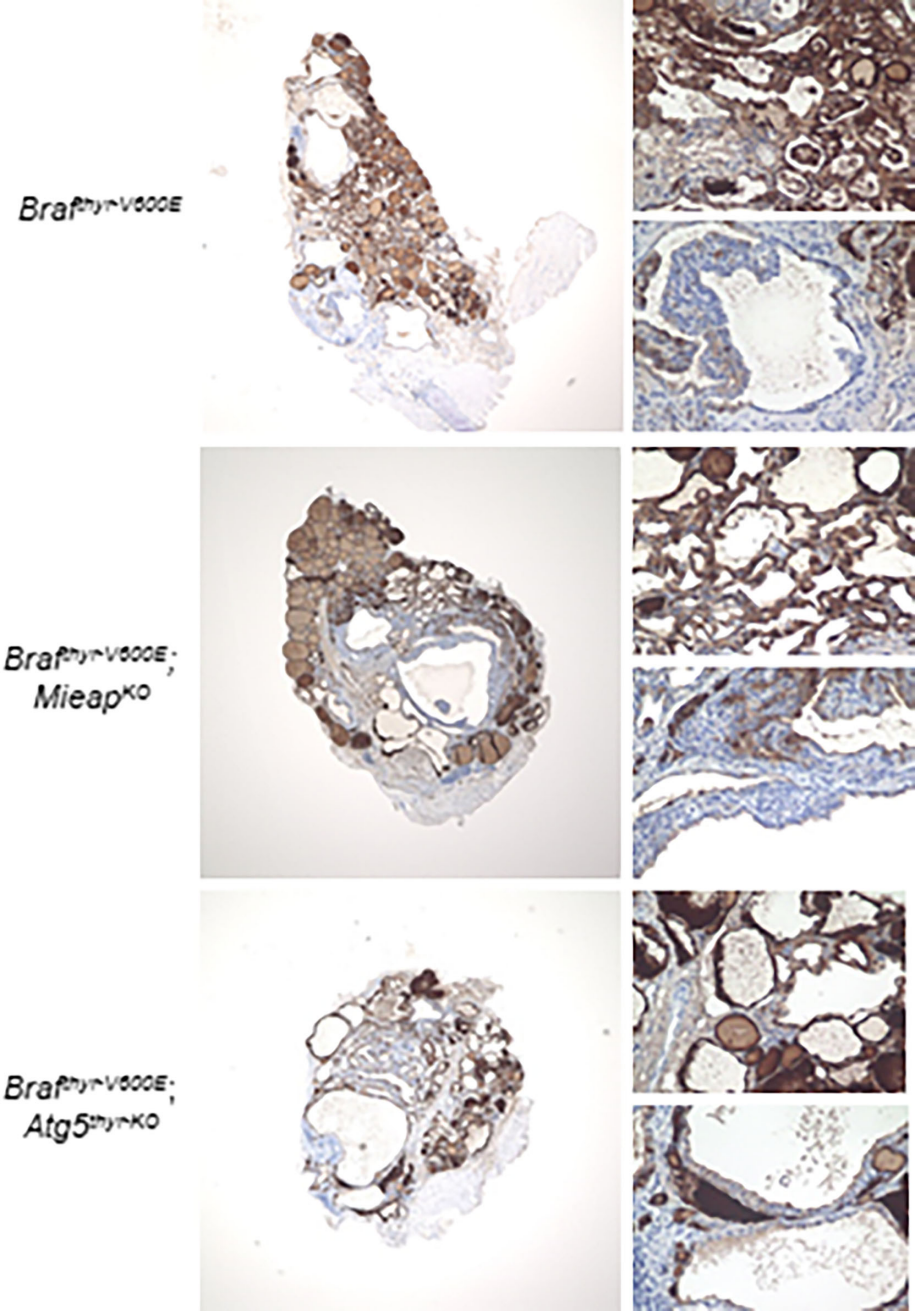
Immunohistochemistry (IHC) staining for thyroglobulin (Tg) expression in the *Braf^thyr–V600E^*, *Braf^thyr–V600E^,Mieap^KO/KO^*, and *Braf^thyr–V600E^,Atg5^thyr–KO/KO^* mice at 12 months. The upper– and lower–right panels in each tissue depict normal and defective Tg expression areas, respectively. The thyroid glands obtained in **Figure 1** were used for Tg staining as described in *Materials and methods*. The original magnifications were ×40 and ×400.

**Figure 3:**
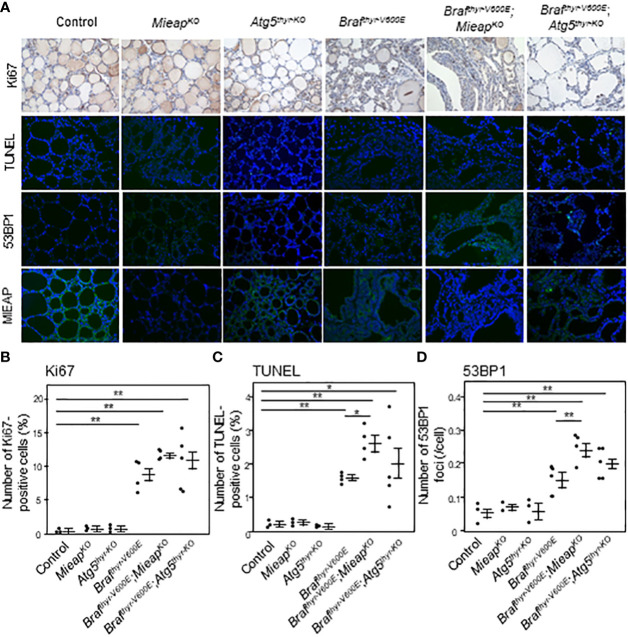
IHC staining for Ki67, TUNEL, 53BP1, and MIEAP expression **(A)** and quantification of Ki67–positive cells **(B)**, TUNEL–positive cells **(C)**, and 53BP1 foci **(D)** in the thyroids from control, *Mieap^KO/KO^*, *Atg5^thyr–KO/KO^*, *Braf^thyr–V600E^*, *Braf^thyr–V600E^*,*Mieap^KO/KO^*, and *Braf^thyr–V600E^*, *Atg5^thyr–KO/KO^* mice at 12 months. The thyroid tissues obtained in **Figure 1** were used for IHC as described in *Materials and methods*. The original magnifications were ×400. Data are shown as black circles for individual mice and as means ± SE. *P < 0.05, **P < 0.01. M, months.

The thyroid tissues obtained at 12 months were examined in detail. All the cancers in the three groups (*Braf^thyr–V600E^*, *Braf^thyr–V600E^*,*Mieap^KO/KO^*, and *Braf^thyr–V600E^*,*Atg5^thyr–KO/KO^* mice) showed comparable Tg expression levels with heterogenous expression patterns (**Figure 2**), indicating that the differentiation status is similar in these groups. The percentages of Ki–67–positive and TUNEL–positive cells, i.e., proliferating and apoptotic cells, respectively, were significantly higher in tumors of the three groups than those of the control (**Figures 3A–C**). The percentages of TUNEL–positive cells in the tumors of *Braf^thyr–V600E^*,*Mieap^KO/KO^* mice were significantly higher than those of *Braf^thyr–V600E^* mice. We also evaluated ROS–induced genomic damages by 53BP1 staining as ROS markers because it has been reported that abnormal mitochondria produce intracellular reactive oxygen species (ROS) (19, 24). 53BP1 accumulates at the DNA double–strand break sites (25). The number of 53BP1 foci was higher in the tumors of the three groups than that of the control (**Figures 3A, D**), and this number in *Braf^thyr–V600E^*,*Mieap^KO/KO^* mice was significantly higher than that in *Braf^thyr–V600E^* mice. Knockout (KO) of MIEAP appears to induce higher ROS than that of ATG5, causing higher 53BP1 foci and also presumably higher apoptotic cells. Finally, BRAF^V600E^ and ATG5 KO did not affect MIEAP expression (**Figure 3A**).

**Figure 4:**
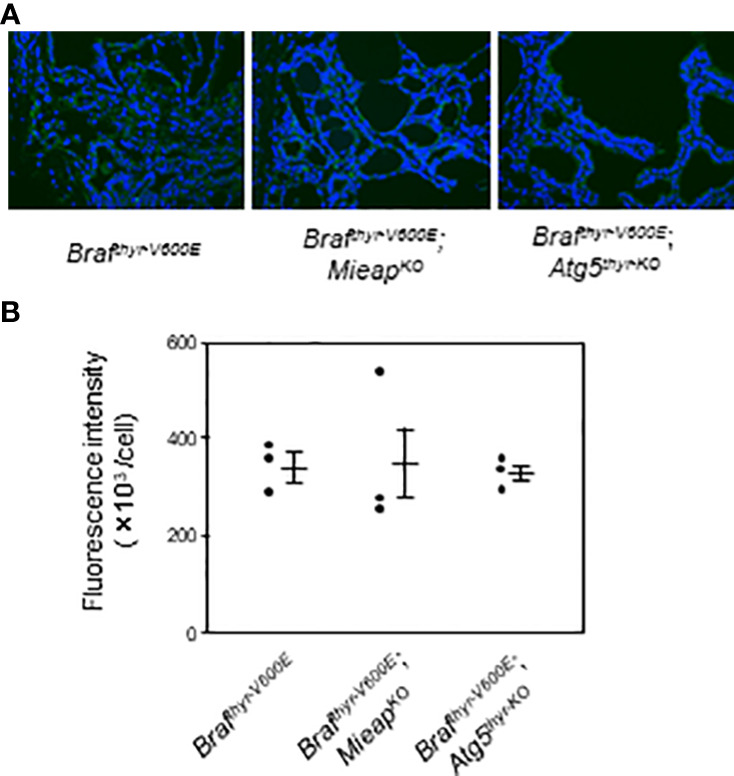
IHC staining for TOMM20 expression **(A)** and quantification of fluorescence intensities **(B)** in the thyroids from *Braf^thyr–V600E^*, *Braf^thyr–V600E^*,*Mieap^KO/KO^*, and *Braf^thyr–V600E^*, *Atg5^thyr–KO/KO^* mice. The thyroid tissues obtained in **Figure 1** were used for IF as described in *Materials and methods*. The original magnifications were ×400. Data are shown as black circles for individual mice and as means ± SE. M, months.

MIEAP and ATG5 play a role in mitochondrial quality control (i.e., elimination of aging, unhealthy mitochondria) as previously reported (24, 26). Therefore, the cytoplasmic accumulation of mitochondria was evaluated by immunostaining with TOMM20. Although the expression was highly heterogenous, the average expression levels were comparable in the three groups (**Figures 4A, B**). Thus, a loss of non–canonical mitophagy (MIEAP KO) or of canonical mitophagy/autophagy (ATG5 KO) by itself is not sufficient for mitochondrial accumulation, that is, the oncocytic phenotype.

Finally, prognostic analysis of MIEAP and ATG5 expression levels using the Human Protein Atlas and TCGA dataset revealed that these two molecules are not prognostic in papillary thyroid cancers (PTCs) (data not shown).

## Discussion

A mitochondrial quality control system, which eliminates abnormal/unhealthy mitochondria, includes canonical mitophagy (a selective type of autophagy) and non–canonical mitophagy. We first examined whether MIEAP, a molecule involved in non–canonical mitophagy, and ATG5, a component of autophagic machinery and involved in canonical mitophagy, are tumor suppressors in thyroid carcinogenesis using genetically engineered mice and found that thyroid cancer development was accelerated in *Braf^thyr–V600E^,Atg5^thyr–KO/KO^* and *Braf^thyr–V600E^,Mieap^KO/KO^* mice compared with *Braf^thyr–V600E^* mice. These results demonstrate that both ATG5 and MIEAP are indeed tumor suppressors and are consistent with previous studies showing an acceleration of development of lung tumors in *lox–stop–lox–Kras^G12D^,Atg5^flox/flox^* or *7^flox/flox^* and *lox–stop–lox–Braf^V600E^,Atg7^flox/flox^* mice intranasally delivered with adenovirus expressing Cre (26–28) and of colon cancers in *Apc^Min^,Mieap^KO/KO^* mice (18). Although the components of the autophagic machinery have multiple functions [including non–selective and selective autophagy and also autophagy–independent functions (29)], mitophagy, a common function of ATG5 and MIEAP, appears to play a role in their tumor–suppressive function. Impairment of mitophagy causes the accumulation of aging, abnormal/unhealthy mitochondria. In this regard, Smith et al. (30) showed accelerated tumor formation with enhanced cell proliferation and reduced apoptosis in a mouse model of colorectal cancers with *Lgr5–creER,Apc^flox/flox^,PolgA^mut/mut^* mice (*PolgA^mut/mut^* mice are a model of accelerated mtDNA mutagenesis) compared with *Lgr5–creER,Apc^flox/flox^* mice. Most of the tumors in the former were complex I–deficient. Furthermore, the growth of adenoma organoids from *Lgr5–creER,Apc^flox/flox^* mice was enhanced by metformin, a complex I inhibitor, confirming that the pharmacological inhibition of complex I can also enhance colon adenoma organoid growth.

In human studies, heteroplasmic germline mtDNA mutations causing complex I dysfunction have shown to shift to homoplasmy in a nasopharyngeal oncocytoma, indicating a selective growth advantage of complex I mutations (31). Omics studies [including genomic (nuclear and mitochondrial) and metabolic landscapes] in renal oncocytoma have indicated that mtDNA mutations are early genetic events, and thereby driver mutations (32). Human colorectal adenoma and adenocarcinomas have been reported to have decreased levels or absence of OXPHOS subunit expression and higher complex I mutations compared to normal tissues (30). In colorectal epithelia, mtDNA mutations causing an OXPHOS defect increase with aging and are enriched during the colorectal tumorigenesis process, suggesting the metabolic benefits of mtDNA mutations in colorectal carcinogenesis. All these data also suggest the important role of mtDNA mutations causing mitochondrial functional defect for tumorigenic process and emphasize the tumor–suppressive function of normal mitochondria.

However, no tumor development in the thyroids of *Mieap^KO/KO^* and *Atg5^thyr–KO/KO^* mice in this study and also in the lungs or colons in the above studies (adenovirus expressing Cre–treated *Atg5/7^flox/flox^* and *Mieap^KO/KO^* mice) suggests that MIEAP or ATG5/7 KO alone is not sufficient for tumor initiation in the thyroids, lungs, and colons. In contrast, benign liver tumor development has been demonstrated in systematic mosaic *Atg5^KO/KO^* and liver–specific *Atg7^KO/KO^* mice (33, 34). Thus, whether the defective expression of certain proteins involved in mitochondrial quality control causing mitochondrial dysfunction can by itself drive cellular transformation or not may be dependent on the tissues examined.

Thyroid cancers developed in *Braf^thyr–V600E^*, *Braf^thyr–V600E^,Atg5^thyr–KO/KO^*, and *Braf^thyr–V600E^,Mieap^KO/KO^* mice are similar in terms of the degree of cell proliferation and differentiated status, but tumor sizes and ROS levels indicated by 53BP1 foci were the highest in *Braf^thyr–V600E^,Mieap^KO/KO^* mice. Thus, mitochondrial damage appear to be higher in *Braf^thyr–V600E^,Mieap^KO/KO^* than *Braf^thyr–V600E^,Atg5^thyr–KO/KO^* mice, suggesting that the role of MIEAP in mitochondrial quality control may be more crucial than that of ATG5.

In addition, we have reported that the expression of MIEAP is suppressed in thyroid conventional cancers in humans (19). However, thyroid cancers in *Braf^thyr–V600^* ^E^ mice in this study preserved MIEAP expression. The mechanism(s) for defective MIEAP in human thyroid non–oncocytic cancers, although unknown, may be specific for humans.

The second purpose of this study was to see whether MIEAP or ATG5 KO causes the oncocytic phenotype in BRAF^V600E^–derived thyroid cancers. As mentioned in the Introduction, we have previously postulated that defective MIEAP expression and increased mitochondrial biogenesis which compensates impaired mitochondrial function are necessary for mitochondrial accumulation (19). Therefore, it is reasonable that the amounts of mitochondria were not increased in thyroid cancers in *Braf^thyr–V600E^,Atg5^thyr–KO/KO^* and *Braf^thyr–V600E^,Mieap^KO/KO^* mice in this study. In contrast, previous studies with mouse models of oncogene–driven cancers have shown that oncogene–derived adenomas did not progress to cancers and were instead diverted to indolent benign oncocytic tumors, indicating the essentiality of autophagy for the progression of benign tumors to a more malignant state (see ref (33). for a review). For example, in the lung cancer models of Cre–activatable *Kras^G12D^* or *Braf^V600E^*, the conditional KO of ATG5 or ATG7 accelerated tumor initiation as mentioned above, which were, however, diverted to benign oncocytoma (26–28). Thus, the conversion of lung adenoma to benign oncocytoma by inhibiting autophagy is considered a potential anticancer strategy. Accumulation of deformed and dysfunctional mitochondria was also observed in liver adenomas in liver–specific *Atg7^KO/KO^* mice mentioned above (34, 35). Again, whether defective mitochondrial function alone can cause the oncocytic phenotype or not may be dependent on the tissues examined.

It should be noted here that, clinically, oncocytomas are usually benign in many tissues, with the thyroid being an exception (36). We could not confirm this difference in this study because thyroid tumors developed in *Braf^thyr–V600E^,Atg5^thyr–KO/KO^* and *Braf^thyr–V600E^,Mieap^KO/KO^* mice were not oncocytic. We need a further study with *Braf^thyr–V600E^,Atg5^thyr–KO/KO^* and *Braf^thyr–V600E^,Mieap^KO/KO^* mice with mtDNA mutations.

In conclusion, we here show that MIEAP or ATG5 KO accelerates the development of thyroid cancers in a mouse model of BRAF^V600E^–mediated thyroid cancer. Although MIEAP or ATG5 KO did not alter the cell proliferation rate or differentiation status in mice and their expression levels are not related to prognosis in human PTCs, our results suggest that these molecules are tumor suppressors. As mentioned above, MIEAP expression is defective not only in oncocytic thyroid tumors but also in conventional thyroid cancers in which BRAF^V600E^ is the main driver mutation, and our results indicate that mitophagy is critical for suppressing carcinogenesis in thyroid cancers in general. However, KO of either molecule alone was not sufficient for the oncocytic phenotype of BRAF^V600E^–mediated thyroid cancers. To establish a mouse model of thyroid oncocytoma, a combination of disruptive mitochondrial function and impaired mitochondrial quality control will be necessary.

## Data availability statement

The original contributions presented in the study are included in the article/supplementary material. Further inquiries can be directed to the corresponding author.

## Ethics statement

This study was reviewed and approved by The Institutional Animal Care and Use Committee at Nagasaki University.

## Author contributions

Conception and design: YN and HA. Administrative support: YN. Provision of study materials: YaN. Collection and assembly of data: KH, TK, and MS. Data analysis and interpretation: KH and YN. All authors contributed to the article and approved the submitted version.

## Funding

This work was supported in part by a Grants–in–Aid for Scientific Research (19K090282) from the Ministry of Education, Culture, Sports, Science and Technology, Tokyo, Japan.

## Conflict of interest

The authors declare that the research was conducted in the absence of any commercial or financial relationships that could be construed as a potential conflict of interest.

## Publisher’s note

All claims expressed in this article are solely those of the authors and do not necessarily represent those of their affiliated organizations, or those of the publisher, the editors and the reviewers. Any product that may be evaluated in this article, or claim that may be made by its manufacturer, is not guaranteed or endorsed by the publisher.

## Abbreviations

ATG, autophagy–related; FA, follicular adenoma; KO, knockout; MIEAP, mitochondria–eating protein; mtDNA, mitochondrial DNA; ROS, reactive oxygen species; Tg, thyroglobulin; TOMM20, translocase of outer mitochondrial membrane 20; 53BP1, tumor suppressor p53–binding protein 1.
